# Adjunctive granisetron therapy in patients with sepsis or septic shock (GRANTISS): Study protocol for a randomized controlled trial

**DOI:** 10.1097/MD.0000000000017354

**Published:** 2019-09-27

**Authors:** Jianbin Guan, Yuexun Guo, Ping Chang, Jianwei Gan, Jian Zhou, Hua Wang, Zhongran Cen, Ying Tang, Zhanguo Liu, Peng Chen

**Affiliations:** a Department of Critical Care Medicine, Zhujiang Hospital; b Department of Pathophysiology, Guangdong Provincial Key Laboratory of Proteomics, Southern Medical University, Guangzhou, China.

**Keywords:** granisetron, protocol, sepsis, septic shock

## Abstract

**Introduction:**

The incidence, mortality, and treatment costs of sepsis are high and, thus, present a major challenge for critical care medicine. Our previous studies suggest that intestinal metabolite granisetron has a potential therapeutic effect on sepsis. Granisetron is a clinically widely used antiemetic, which is safe, inexpensive, and reliable. However, its value in the treatment of sepsis remains unclear. This study aims to explore the efficacy and safety of granisetron in the treatment of sepsis.

**Methods and analysis:**

A single-center, single-blind, randomized, controlled clinical trial will be conducted on 154 patients with sepsis. Patients who meet sepsis 3.0 diagnostic criteria, aged ≥18 and ≤80 years, with PCT ≥ 2 ng/mL will be recruited. Patients will be randomized to receive intravenous granisetron 3 mg every 8 hours (n = 77) or an equal volume of normal saline (n = 77) for a treatment period of 4 days or to ICU discharge. The primary outcome is 28-day all-cause mortality. Secondary outcome measures include requirements for organ function support, changes of organ function, changes in infection biomarkers, changes in inflammatory and immune biomarkers, and the proportion of new organ failure. Adverse events and serious adverse events also will be observed closely.

**Ethics and dissemination:**

The study was approved by the Clinical Ethics Committee of Zhujiang Hospital of Southern Medical University (2018-ZZJHZX-009). The trial results will be disseminated at national and international conferences and through peer-reviewed journal.

**Trial registration:**

NCT03924518.URL: www.clinicaltrials.gov.

**Protocol date:**

1 May 2019. version 2.1.

## 1 Introduction

Sepsis is life-threatening organ dysfunction due to a dysregulated host response to infection and has a high mortality rate.^[1,2]^ It is recognized as a primary health threat.^[3]^ Epidemiological studies have shown that 250,000 of the 750,000 patients who suffer from sepsis each year in the United States die as a result of the disease. Treatment costs for sepsis are high. The average cost per patient with sepsis in the United States is $22,000 and about ¥80,000 in China.^[4,5]^ Even for those who survive, the quality of life is significantly worse.^[6]^

At present, the internationally recognized management strategies for patients with sepsis mainly include early active fluid resuscitation, early appropriate antibiotics, hemodynamic support of vasopressin, and identification and control of infection sites.^[7]^ The implementation of the above treatment strategies has caused the prognosis of patients with sepsis to be significantly improved. However, sepsis is a life-threatening organ dysfunction caused by infection. A widespread and uncontrolled immune inflammatory response results in its development and poor prognosis. Although the use of antimicrobials, fluid resuscitation, and organ function support have certainly improved the prognosis, to date, the mortality of sepsis continues to be on the range of 20% to 30%, and in developing countries it is as high as 60%.^[8,9]^ Therefore, it is necessary to find a safe, reliable, and inexpensive new treatment strategy that can reduce mortality further. Recent studies have shown that changes in metabolites produced by gut microbes play a role in the development of sepsis and organ damage. The number of intestinal microbes in normal humans is as high as 10^12^ to 10^14^.^[10]^ The intestinal flora participates in important physiological processes such as nutrient absorption, substance metabolism, and immune defense.^[11]^ Our previous animal studies have confirmed that there is a significant deficiency of intestinal flora metabolites called granisetron in sepsis sensitive mice. This deficiency is closely related to organ dysfunction and death.^[12]^ Administration of granisetron to septic mice has shown to significantly reduce the damage in their liver and lungs, while reducing the mortality rate.^[12,13]^ Subsequently, we also found that in septic patients, fecal granisetron level is negatively related to liver damage.^[12]^ Based on the above findings, we hypothesized that endogenous granisetron deficiency has a significant impact on poor prognosis in patients suffering from sepsis.

Granisetron is a highly selective 5- hydroxytryptamine 3 (5-HT3) receptor antagonist that has been widely used clinically to treat vomiting after chemotherapy with mild side effects, high safety, and low cost.^[14]^ Theoretically, for patients with sepsis, supplementation with granisetron may be an effective treatment for reducing organ damage and improving the survival rate of patients. However, there is no clinical evidence for whether granisetron can greatly improve the prognosis of patients with sepsis than a placebo. We, therefore, designed a study to evaluate the efficacy and safety of granisetron treatment compared with placebo in patients with sepsis.

## 2 Methods

The protocol and analysis plan have been reviewed by the Clinical Trial Committee of Zhujiang Hospital (2018LX0003-GY). Patients and the public were not involved in the design of this study. We report our protocol for the GRANTISS trial based on the SPIRIT guidelines.^[15]^

### 2.1 Design

The GRANTISS trial is single-center, single-blind, randomized, and placebo-controlled. It is expected to be conducted in the Department of Critical Care Medicine of Zhujiang Hospital from April 23, 2019 to December 31, 2020. The hospital, a large-scale research tertiary hospital in Guangzhou, China, provides treatment mainly to patients from various provinces of South China, covering a wide range; this ensures, to some extent, that the patients recruited are widely representative. The study ends with the last patient follow-up.

Eligible patients will be randomized and placed in the granisetron treatment group or the placebo control group, and all enrolled patients will receive routine standard care and standardized treatment. On this basis, patients in the granisetron-treated group will receive granisetron intravenously, and patients in the placebo-controlled group will receive the same volume of saline.

### 2.2 Population

Any patients who are suspected of sepsis or have been confirmed sepsis in intensive care unit (ICU) will be potential candidates. However, we will only include patients meeting the following criteria: age ≥18 years and ≤80 years of age; meet the 2016 International Sepsis Guidelines diagnostic criteria: suspected or confirmed infection and evidence of acute organ dysfunction: for patients without chronic organ dysfunction, sequential organ failure assessment score (SOFA) ≥ 2; for patients with chronic organ dysfunction, SOFA increased by ≥ 2; and procalcitonin (PCT) ≥ 2 ng/mL.

Patients will be excluded if they meet any of the following criteria: age<18 years old or > 80 years; pregnancy or lactation; solid organ or bone marrow transplant; myocardial infarction within the past 3 months; advanced pulmonary fibrosis; cardiopulmonary resuscitation before enrollment; HIV-positive; neutropenia; hematological/lymphatic tumors have no remission; limited care (lack of commitment to full and aggressive support); long-term use of immunosuppressive drugs or immunodeficiency; advanced tumors; combined with noninfectious factors leading to death (uncontrolled large bleeding, cerebral hernia, etc.); persistent infection sources that cannot be removed by puncture and drainage, debridement, or other surgical procedures; allergy to granisetron; intestinal obstruction.

### 2.3 Screening and informed consent

Two highly trained screeners will screen for all sepsis patients who meet the criteria. Only patients who have been confirmed by both screeners as meeting the inclusion criteria and who do not meet any of the exclusion criteria will be eligible. If 2 screeners have different opinions on the eligibility of the patients, it will be judged by the principal investigator. After determining that the patient is eligible for inclusion and discussing the risks associated with the treatment with the treating physicians, the screeners need to inform the patients or their families of the purpose of the trial and its risks; then the patients or their relatives must sign the informed consent. For successful enrollment, the screeners should register their enrollment date and basic information in detail. For excluded patients, the screeners should record the reason for the exclusion in detail. During the study period, the primary investigator should conduct a random check on the screening form every month to ensure that all enrolled patients meet the criteria. The screening form will be permanently stored in paper and spreadsheet forms.

### 2.4 Randomization allocation concealment

Patients who are eligible for enrollment will be randomized, and they will be assigned with an equal chance to the granisetron and placebo control group, with a 1:1 ratio between the treatment and control groups. The random sequence was generated by an independent Data Security Management Board (DSMB) using Stata 13, and the randomized cards were made by the relevant personnel according to the random sequence. Each random group card was marked with a patient's enrollment group and loaded into a customized sealed envelope. The randomly grouped block lengths will not be made public until the trial is completed. After successful recruitment, the clinical screeners will open the envelope and complete the random assignment based on the randomized information. If more than 1 patient is successfully recruited at the same time, the order of randomization for patients is determined based on the registered time of ICU admission.

### 2.5 Blinding

The trial used a single-blind design in which the subjects, subject relatives, and relevant statisticians will remain blind to the trial grouping. To achieve this process, the control group will use normal saline as a placebo with the same appearance as granisetron and use same 50 mL syringes as drug containers and use the word “GRAN” as the drug infusion label.

### 2.6 Interventions

The recruited patients will begin the trial intervention within 8 hours after randomization. Patients in the granisetron-treated group will receive granisetron 3 mg (PKU HealthCare Corp, Ltd) diluted to 25 mL with 22 mL of saline and intravenously administered within 10 minutes. Patients in the placebo group will receive 25 mL of normal saline and administer it intravenously within 10 minutes. The trial intervention will be administered at a frequency of 8 hours, with a course of treatment of 4 days or ICU discharge, whatever comes first. Considering the fluctuation of vital signs such as body temperature and blood pressure at different time points in 1 day, to avoid the influence of the abovementioned confounding factors on the trial results, it is stipulated that the trial intervention for each patient will be held at 12:00, 20:00, and 04:00. In addition to the trial intervention, patients will receive routine standard care and routine sepsis treatment according to the 2016 International Management Guidelines for Sepsis,^[7]^ including but not limited to the use of antibiotics, venous fluid resuscitation, use of blood products, enteral or parenteral nutrition, prevention of deep vein thrombosis, and supportive treatment for organ dysfunction. All treatments except study interventions will be determined by the treating physician and will be noted in detail in patients’ medical records.

For patients who may require using antiemetics, any antiemetics that act on 5-HT receptors should be avoided, but other classes of antiemetics such as metoclopramide may be used. Additionally, in the treatment of patients with sepsis, some liver-protective drugs such as reduced glutathione are often used to reduce septic liver damage; because the role of granisetron for liver function protection in clinic is not certain, the use of such drugs to alleviate organ dysfunction by the treating physician will be allowed, but the name, frequency, and dosage of the drug used should be accurately recorded.

### 2.7 Data collection

The patients enrolled in the group will receive clinical or laboratory assessments (Table 1) during the screening period, the drug infusion period, on the first day after the end of trial interventions, and on the 28th day after enrollment. Before treatment (D0; screening period), the following indicators should be evaluated: pathogen culture results, the level of blood 5-HT, acute physiology and chronic health evaluation (APACHE II) scores, and SOFA scores. Before treatment (D0; screening period), on the third day of treatment (D2), and on the first day after treatment (D4), the following indicators will be measured: immune function indicators (white blood cell count, peripheral lymphocyte count, CD4+ and CD8+ T-cell count), renal injury indicators (Cys C), inflammation Indicators (the level of IL-6, CPR, ESR, SOD), and infection indicators (the level of serum PCT, fungi (1,3)-beta-D-Glucan, galactomannan antigen). Daily assessment indicators include the following: liver injury indicators (the level of ALT, AST, total bilirubin, direct bilirubin), the level of blood lactate, renal injury indicators (the level of Cr, BUN), lung injury indicators (oxygenation index, not applicable during extracorporeal membrane oxygenation (ECMO) support), fluid balance, vasoactive drug dose, urine volume, SOFA scores, and concomitant medications that may affect the outcome. Other indicators are the duration of organ function replacement therapy including continuous renal replacement therapy (CRRT), ECMO and mechanical ventilation, the incidence of new dysfunction, ICU stay time, ICU mortality, and 28-day mortality. Safety indicators include adverse reactions and the incidence of serious adverse events. Among them, the 28-day survival information of patients will be obtained by telephone follow-up. For dropout events, the causes will be recorded in detail and these patients will also be followed up. All the laboratory outcomes will be taken from the Central Laboratory of Zhujiang Hospital, and all the indicators needed to calculate the APACHE II score and SOFA score will also be recorded in detail. Furthermore, during the course of treatment, any out-of-plan evaluations of the above indicators by the treating physicians also will be collected. All of the above data will be collected by highly trained clinical observers.

**Table 1:**
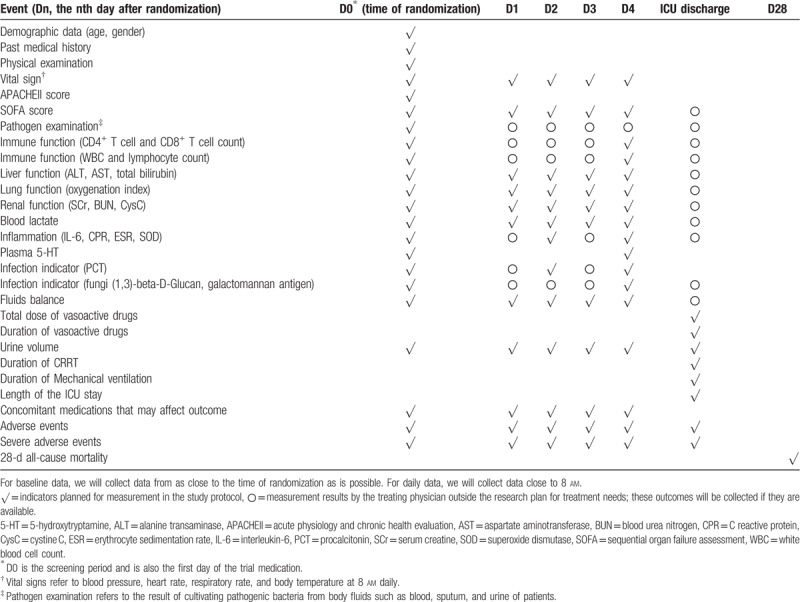
Schedule of clinical or laboratory assessments.

A specially designed case report form (CRF) will be used for data collection. The data will be collected by clinical observers. One observer will record the data first, and at least one observer will check the recorded data. The data is then recorded on web-based electronic CRFs on www.cloud.medsci.cn. To protect patient privacy, the CRF tables will only record patient initials and enrollment numbers as identifiers.

### 2.8 Outcome measurement

#### 2.8.1 Primary outcome

The primary outcome measure is the all-cause mortality rate at 28 days after enrollment.

#### 2.8.2 Secondary outcome

The secondary outcome indicators mainly include the following aspects:

Requirements for organ function support: the duration of organ function replacement therapy including CRRT, ECMO, and mechanical ventilation
Improvement of organ function: the changes in SOFA score; creatinine, transaminase, and cystatin after interventions completion; lactate clearance after interventions completion; and the incidence of new organ dysfunction
Improvements in infection indicators: the changes in PCT after interventions completion, PCT clearance after interventions completion, and changes in fungi (1,3)-beta-D-Glucan and galactomannan antigen after interventions completion.
Improvement of inflammatory indicators: the changes in IL-6, ESR, SOD, and CRP after interventions completion
Improvement of immune function indicators: the changes in lymphocyte count, white blood cell count, and CD4+ and CD8+ T cell count after interventions completion.
The incidence of new organ dysfunction: the incidence of new acute liver injury, kidney injury, and lung injury. New organ failure refers to organ failure that occurs after enrollment.
Other indicators: ICU length of stay (LOS).
Additionally, we will also collect blood 5-HT levels before and after completion of the treatment to determine if there is a correlation between 5-HT levels and the prognosis of sepsis.

#### 2.8.3 Adverse events and serious adverse events

Granisetron has mild side effects in healthy people. Common side effects include headache (10%–15%), constipation (2%–4%), lethargy (2.5%–5%), diarrhea (0.6%–2%), and dizziness (0.5%–3.2%).^[14]^ According to reports, the 5-HT3 receptor inhibitor may also prolong the QT interval of the heart, but this is very rare.^[16]^ However, reports of side effects in granisetron in critically ill patients are lacking. Therefore, this study will also focus on the possible adverse effects of using granisetron for patients with sepsis. Observers will dynamically record treatment-related side effects daily, including headache, constipation, bloating, diarrhea, nausea, vomiting, arrhythmia, and other side effects that treatment physicians believe are related to the study. The time and frequency of the side effects should be recorded in detail and reported to a specialized expert group on side effects, which consists of three senior physicians dealing with critically ill patients, who will judge the correlation between adverse events and research interventions.

A serious adverse event is any event that meets the following criteria within 28 days of the start of the study: results in death; is life-threatening; results in prolongation of the patient's hospitalization period; or results in a persistent or significant disability.

Any serious adverse events that occur during the trial should be recorded. If it is determined as relevant to the study, the study interventions will be immediately stopped, and the necessary treatment for serious adverse events will be taken and immediately reported to the Ethics Committee. Researchers in the GRANTISS study group and all the treating physicians should undergo training prior to the start of the study to be able to accurately identify serious adverse events.

### 2.9 Study flowchart

**Figure 1:**
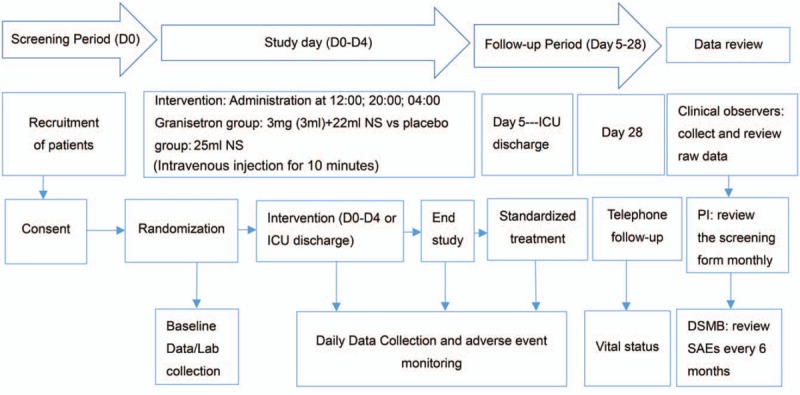
The flow diagram of the GRANTISS study.

The study flowchart is presented in Fig. 1.

### 2.10 Data and safety monitoring

An independent DSMB comprised of 3 clinical experts, 1 competent nurse, and 1 statistician will monitor the progress and safety of the trial. When the trial is actively recruiting, the DSMB will be responsible for reviewing nonintervention data every 6 months, including the implementation of standard care measures and conventional treatments, dropout events, adverse events, and serious adverse events. The committee has the right to suspend the trial at any point to ensure that it is implemented scientifically and in an ethically appropriate manner.

### 2.11 Sample size

According to previous clinical experience, the mortality rate of sepsis in this study center is about 33%. Our sample size was calculated to detect a 20% difference in mortality on day 28 between the 2 groups with a 2-tailed test, a = 0.05, β = 0.2 (power = 80%). Considering some factors such as shedding and loss of follow-up, an additional 15% of the sample size was added, resulting in a total of 154 patients, 77 in the control group, and 77 in the treatment group.

### 2.12 Statistical analysis

Statistical analysis plan has been reviewed and approved by professional medical statisticians from the Clinical Trial Committee of Zhujiang Hospital (2018LX0003-GY).

#### 2.12.1 Statistical analysis data set

The analysis will be based on a modified intent-to-treat population in which patients who have undergone at least 1 study intervention and one assessment can be included in the analysis.

#### 2.12.2 Basic statistical principles

All statistical inferences will be performed using a 2-sided test unless otherwise specified. The statistically significant test level is at 0.05, and the interval estimate for the parameters used a 95% confidence interval. Statistical inference will be performed using the parameter method as much as possible. When the data does not satisfy the parameter method condition, the data conversion method can be used to satisfy the condition. If it is still not satisfied, a nonparametric method can be considered.

#### 2.12.3 Processing of missing data

Analysis based on modified intent-to-treat population sets using the last observation carried forward method, that is, the case where the entire treatment process is not observed but rather the last observation data is transferred to the final result of the test

#### 2.12.4 Shedding analysis

The total shedding rate of each group and the comparison of shedding due to adverse events will be calculated using the Pearson chi-square test.

#### 2.12.5 Statistical description

The measurement data will be used to calculate the mean, standard deviation, interquartile range, median, and so on. Count and rank data will be calculated for frequency and composition ratio, as well as median and average rank. Qualitative data will be given the positive rate, the number of positive, and denominator cases.

#### 2.12.6 Analysis of baseline data

It includes demographic indicators, preintervention general conditions, and laboratory indicators. The measurement data will be tested by *t* test or *t*’ test (heterogeneity of variance); the count data will be assessed using Pearson chi-square test; and the rank data is tested by Wilcoxon rank sum test.

#### 2.12.7 Compliance analysis

Compliance analysis will be performed according to the dose and time of administration.

#### 2.12.8 Primary outcome

The 28-day all-cause mortality difference will be compared using Pearson chi-square test and logistic regression analysis with adjustment for the variables may affect mortality rate.

#### 2.12.9 Secondary outcome

Quantitative variables will be compared between groups using a 2-sample *t* test. Repeated measures variables will be compared between groups using covariance analysis or mixed linear model analysis. The Pearson chi-square test will be used for qualitative variables, and the Wilcoxon rank sum test will be used for grading variables.

#### 2.12.10 Survival analysis

If the 60-day survival information of patients is obtained, the time of death in the 60-day survival of the 2 groups of patients will be described as Kaplan–Meier plots, and survival differences will be tested using the Cox proportional hazard model.

#### 2.12.11 Subgroup analysis

APACHE II score >25 versus ≤25, age >65 versus ≤65, time of sepsis diagnosis at recruiting >48 vs. ≤48 hours, multiple organ dysfunction versus nonmultiple organ dysfunction patients will be taken as influencing factors for the subgroup to analyze and calculate the interaction of interventions with subgroup factors. The relative risk value of each group and the 95% CI interval, the number of events/total number will be given in the form of a forest map.

#### 2.12.12 Safety analysis

The incidence of adverse events will be compared using the chi-square test. The adverse events that occur in the trial will be listed. For the quantitative indicators, the corresponding difference test method will be used to compare between groups or compare within groups.

#### 2.12.13 Interim analysis

No interim analysis was scheduled.

### 2.13 Protocol amendments

Any changes to the protocol that may affect the outcome of the trial, potential benefits, and safety of patients including test purpose, test design, test population, and sample size changes should be submitted to the Ethics Committee and the Clinical Trial Center in a written report. It can be implemented only after obtaining the consent of the above 2 departments.

## 3 Discussion

Granisetron is widely used in clinical practice as a central antiemetic. Its pharmacological effect is mainly to inhibit the effect of 5-HT by inhibiting the 5-HT3 receptor. 5-HT can cause the release of a large number of inflammatory mediators during sepsis and can also mediate oxidative stress and lipid peroxide.^[17–19]^ Eventually, vascular endothelial cells are damaged and tissue permeability increased.^[20]^ We have previously found beneficial effects of granisetron on sepsis. In sepsis animal models, we found septic mice with low granisetron levels were more likely to die and that supplementation of exogenous granisetron protected mice against cecal ligation and puncture-induced death and liver and lung injury.^[12,13]^ In patients with sepsis, there is a significant negative correlation between the levels of fecal granisetron and the degree of septic liver injury, suggesting that granisetron may be a new approach to reduce organ damage and improve prognosis in patients with sepsis. Granisetron is a safe, inexpensive, and clinically available antemetic and can be transferred quickly to sepsis treatment once the beneficial efficacy is confirmed. Therefore, we planned to conduct this clinical study on granisetron in the treatment of sepsis to initially explore the safety and efficacy of this potential therapeutic approach.

## 4 Ethics and Dissemination

The study was approved by the Clinical Ethics Committee of Zhujiang Hospital of Southern Medical University (2018-ZZJHZX-009). The research team will ensure that this study is carried out in accordance with the principles of the Helsinki Declaration.^[21]^

We will submit the primary and secondary results for publication to a peer-reviewed journal or disseminate them at national and international conferences.

## Acknowledgment

The authors thank Peihua Cao for guidance on statistical analysis protocol. Dr Peihua Cao is a medical statistics expert in Clinical Trial Committee of Zhujiang Hospital, Southern Medical University, Guangzhou, China.

## Author contributions

**Conceptualization:** Peng Chen, Zhanguo Liu, Ping Chang.

**Data curation:** Jianbin Guan, Yuexun Guo, Jianwei Gan, Ying Tang, Jian Zhou, Zhongran Cen.

**Formal analysis:** Jianbin Guan, Yuexun Guo, Jianwei Gan.

**Methodology:** Peng Chen, Zhanguo Liu, Jianbin Guan, Yuexun Guo.

**Project administration:** Peng Chen, Zhanguo Liu, Ping Chang.

**Resources:** Zhanguo Liu, Ping Chang.

**Supervision:** Peng Chen, Zhanguo Liu, Ping Chang.

**Writing – original draft:** Jianbin Guan, Zhanguo Liu.

**Writing – review & editing:** Peng Chen, Zhanguo Liu, Ping Chang, Jianbin Guan, Yuexun Guo, Jianwei Gan, Ying Tang, Jian Zhou, Zhongran Cen, Hua Wang.
